# Management of a Complex Medial‐End Clavicle Fracture With Hook Plate Fixation After Failed Prior Surgeries: A Case Report and Narrative Review of Surgical Options

**DOI:** 10.1155/cro/5764986

**Published:** 2026-04-30

**Authors:** Hyung-Seok Park, Jong-Hyeon Nam, Jeong-Soo Oh

**Affiliations:** ^1^ Department of Orthopedic Surgery, Chosun University Hospital, Gwangju, Republic of Korea, chosun.ac.kr; ^2^ Department of Orthopedic Surgery, College of Medicine, Chosun University, Gwangju, Republic of Korea, chosun.ac.kr

## Abstract

Medial‐end clavicle fractures are rare, and the published literature is limited. Most injuries can be managed nonoperatively with favorable outcomes; however, displaced or comminuted fractures, symptomatic delayed union/nonunion, or fixation failure may require surgery, and operative management is challenging because of the proximity to vital mediastinal structures. We describe a 38‐year‐old man with a left medial‐end clavicle fracture sustained in a fall from approximately 9–10 m. Two prior plate fixation attempts at an outside clinic failed to maintain stable reduction, with persistent pain and fracture‐site prominence. At our facility, computed tomography confirmed an intact sternoclavicular joint but revealed severe comminution at the proximal fracture site with anterosuperior displacement of the distal fragment. We removed the failed hardware and performed revision fixation using a clavicle hook plate, intentionally limiting screw purchase to the distal fragment to reduce mediastinal risk. Postoperatively, teriparatide was administered as an adjunctive therapy because of concern for impaired healing after repeated fixation failure. The implant was removed on 28 October 2024 after radiographic union, and at the final follow‐up on 18 April 2025, union was maintained and pain was minimal (VAS ≤ 1/10); standardized functional outcome scores were not available. This single case suggests that hook plate fixation may be a salvage option when safe medial screw purchase is not feasible; interpretation is limited by the case‐report design and adjunctive pharmacologic therapy.

## 1. Introduction

Medial‐end clavicle fractures are uncommon, reported to account for less than 5% of clavicle fractures in adults, and frequently result from high‐energy mechanisms [1, 2]. Owing to their rarity, guidance on optimal management is limited, and many nondisplaced or stable injuries are treated nonoperatively with favorable outcomes [2, 3]. Operative treatment may be indicated for severe displacement or comminution, open injury, neurovascular compromise, symptomatic delayed union/nonunion, or fixation failure [4–6]. Decision‐making is further complicated by the proximity of the medial clavicle to critical mediastinal structures—including the great vessels, trachea, esophagus, brachial plexus, and apical pleura—highlighting the need for careful imaging and surgical planning [6–8]. When the medial bone stock is insufficient, standard locking‐plate constructs may not allow safe medial screw purchase; alternative strategies, including contoured locking plates in selected patterns and clavicle hook plates, have been reported with acceptable outcomes in small series [7, 9, 10]. However, reports describing hook plate fixation as a salvage strategy after failed prior surgeries are scarce. Here, we present a revision case treated with hook plate fixation and provide a narrative, selective review of surgical options for complex medial‐end clavicle fractures.

## 2. Case Presentation

The patient was a 38‐year‐old man who fell from a height of 9–10 m, sustaining a left medial‐end clavicle fracture. He was a nonsmoker and had no chronic comorbidities. At the time of injury, he also sustained a traumatic intracranial hemorrhage and was managed according to neurosurgical trauma protocols. No associated thoracic, shoulder girdle, or chest wall injuries were identified. Initial imaging demonstrated a displaced, comminuted medial‐end fracture, and the outside clinic elected operative fixation. After two unsuccessful operations at the outside clinic, he presented to our hospital with persistent pain and visible prominence at the fracture site. Review of outside radiographs suggested recurrent loss of reduction after plate fixation attempts. Given the severe comminution of the proximal fragment, inadequate screw purchase may have contributed to the fixation failure. At presentation, sternoclavicular joint (SCJ) stability was assessed clinically (no abnormal mobility on gentle examination) and radiographically. Computed tomography demonstrated a congruent SCJ without dislocation; there was marked comminution at the medial (proximal) fracture site and anterosuperior displacement of the distal fragment (Figure 1).

Figure 1Initial injury imaging (2024‐02‐27). (a–b) Anteroposterior (AP) radiographs demonstrate medial‐end comminution with anterosuperior displacement of the distal fragment; the sternoclavicular joint (SCJ) remains aligned. (c) 3D CT reconstruction of the thoracic inlet shows a medial‐end fracture of the left clavicle.

**Figure:**
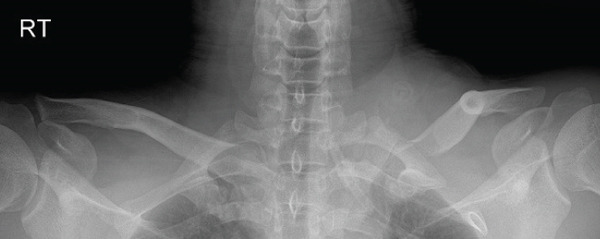
(a)

**Figure:**
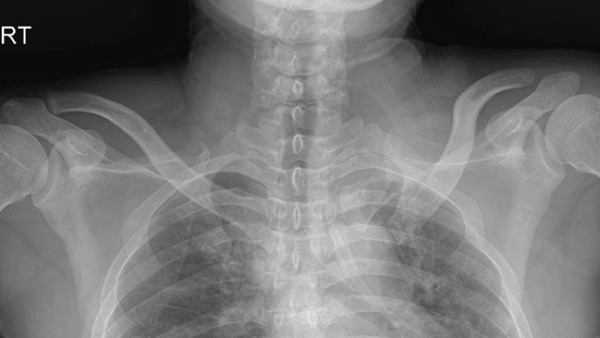
(b)

**Figure:**
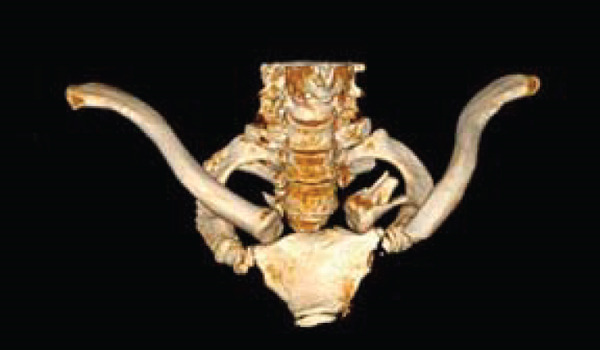
(c)

### 2.1. Diagnostic Reasoning

The primary working diagnosis was a displaced medial‐end clavicle fracture with persistent mechanical symptoms after fixation failure. Differential considerations included SCJ dislocation/subluxation, symptomatic nonunion or malunion, isolated implant failure without ongoing fracture instability, and postoperative infection. SCJ instability was considered because medial clavicle injuries can mimic dislocation clinically; however, clinical examination showed no abnormal SCJ translation on gentle testing and computed tomography demonstrated a congruent SCJ without dislocation. Infection was considered given the history of repeated surgery, but there were no clinical signs of deep infection (e.g., wound drainage, erythema, or systemic symptoms), and imaging findings were most consistent with mechanical instability in the setting of severe comminution and inadequate medial bone stock.

Given the complex comminution, persistent symptoms, and prior fixation failure, we planned removal of the previous hardware and revision fixation. Because medial screw purchase was considered unsafe due to limited proximal bone stock and proximity to mediastinal structures, we selected a hook plate as a salvage option. To minimize mediastinal risk, the plate was positioned such that screw purchase was confined to the distal fragment (Figure 2).

Figure 2Local surgeries. (a) AP radiograph after the first operation (2024‐03‐07). (b) AP radiograph after the second operation (2024‐04‐20). (c–d) Axial CT after the second operation.

**Figure:**
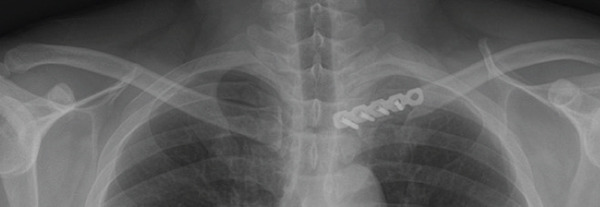
(a)

**Figure:**
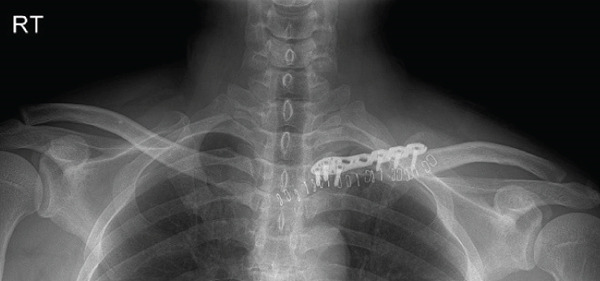
(b)

**Figure:**
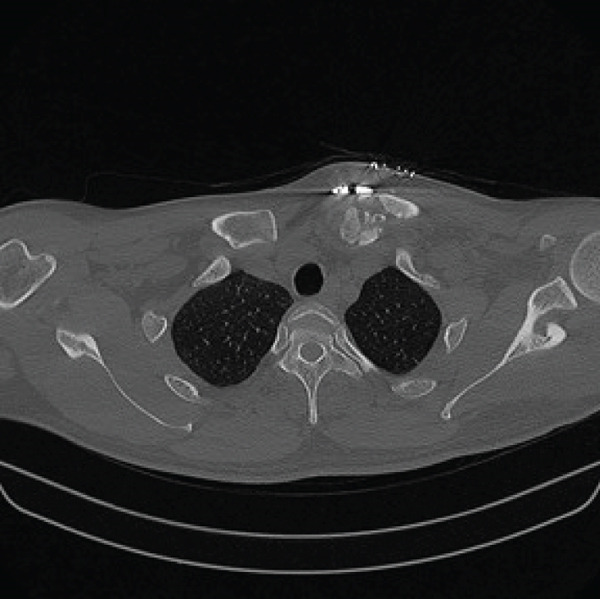
(c)

**Figure:**
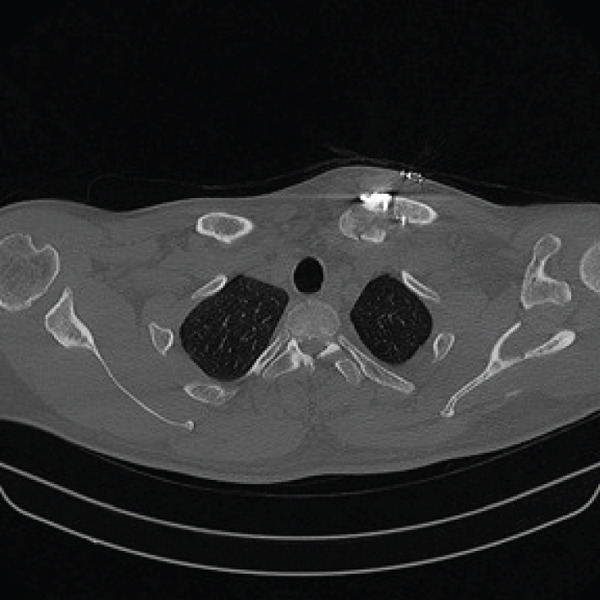
(d)

## 3. Operative Details and Postoperative Course

### 3.1. Operative Setup and Technique

The patient was positioned supine with a small bump between the scapulae to elevate the chest. The procedure was performed through the prior medial‐end clavicle incision. After careful subperiosteal exposure and removal of the failed implants, the fracture site was debrided and reduced. A 6‐hole clavicle hook plate (Synthes, Solothurn, Switzerland) was contoured to fit the medial clavicle; the hook was gently seated at the manubrial (sternal) side near the sternoclavicular region under fluoroscopic guidance (AP and cephalad‐tilt views) to confirm position. To reduce mediastinal risk, screw fixation was confined to the distal fragment; three 3.5‐mm cortical screws were inserted (22 mm × 2 and 24 mm × 1) after measuring with a depth gauge under fluoroscopy. Postoperative radiographs confirmed restored alignment, and the patient reported symptomatic improvement (Figure 3).

Figure 3Immediate postoperative radiographs at our hospital (hook plate revision). (a–b) AP/cephalad radiographs demonstrate restored alignment and stable fixation with screw purchase limited to the distal segment.

**Figure:**
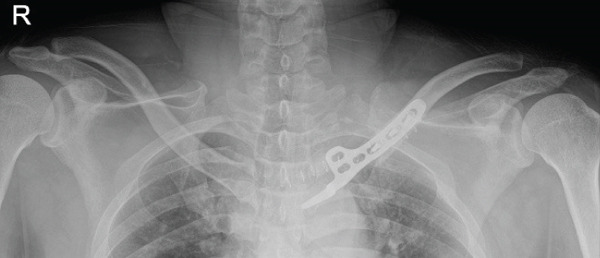
(a)

**Figure:**
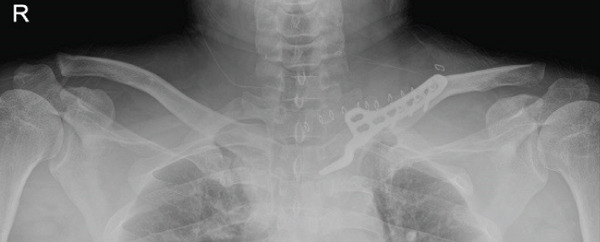
(b)

### 3.2. Mediastinal Safety and Hook Depth Control

Given the proximity of the great vessels and other mediastinal structures, exposure was kept strictly subperiosteal with minimal posterior dissection. The hook was advanced under direct tactile feedback with its tip kept in contact with the manubrial cortex; a shallow seating position was favored to avoid deep penetration. Fluoroscopy (AP and cephalad‐tilt views) was used to confirm that the hook remained appropriately seated at the sternal side and did not project beyond the posterior cortex.

### 3.3. Screw Length and Trajectory

Screw lengths were selected using a depth gauge. When drilling, a guarded drill technique (drill stop) and oblique fluoroscopic checks were used to reduce the likelihood of posterior cortical penetration. Screw trajectories were oriented to maximize purchase within the distal fragment while avoiding posterior protrusion toward the mediastinum; final lengths (22 mm × 2 and 24 mm × 1) were confirmed under fluoroscopy.

### 3.4. Intraoperative SCJ Stability Assessment

SCJ stability was assessed before and after fixation by gently translating the medial‐end clavicle anteriorly and posteriorly relative to the manubrium and comparing with the contralateral side. No gross instability was appreciated. Fluoroscopic assessment confirmed the maintenance of SCJ congruity after hook seating and final screw fixation.

### 3.5. Adjunctive Bone Anabolic Therapy

Because of concern for impaired healing after repeated fixation failure and severe comminution, teriparatide (PTH 1‐34; Forsteo) was prescribed as adjunctive therapy (off‐label for fracture healing). Teriparatide 20 *μ*g was administered once daily starting on postoperative Day 1 and continued for approximately 4 months (until 9 August 2024).

**Figure 4:**
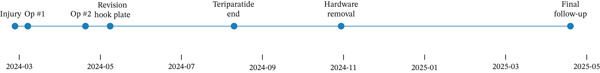
Clinical timeline (schematic). Schematic timeline from injury to prior operations, revision fixation, adjunctive teriparatide use, union, implant removal, and final follow‐up.

A chronological summary of the clinical course is provided in Table 1 and depicted in Figure 4. The postoperative rehabilitation protocol is summarized in Table 2.

## 4. Discussion

Since medial‐end clavicle fractures are uncommon and adjacent to critical mediastinal structures, consensus on optimal management is limited [2, 3, 11]. Many nondisplaced or stable injuries fare well with nonoperative care, but surgery may be appropriate for displaced or comminuted patterns and after fixation failure [12, 13]. In the published literature, a variety of operative constructs have been described (e.g., contoured locking plates, hook plates, spanning constructs, and ligament reconstructions), typically in small case series. This discussion and Table 3 represent a narrative, nonsystematic summary of selected reports; outcome measures, follow‐up duration, and reported union/complication rates vary across studies, and no direct comparisons are intended. When adequate medial bone stock allows safe screw purchase, locking‐plate constructs have been used with reported union and symptom improvement in some series [9, 14]. Hook plates have also been reported when medial purchase is limited, with outcomes ranging from uneventful union to implant‐related irritation and manubrial changes requiring planned removal [10]. In our patient, two prior plate fixations failed to maintain stability. While the precise mechanism of failure cannot be proven from retrospective review alone, severe medial‐end comminution likely limited screw purchase in the proximal fragment and may have contributed. As a salvage strategy, we used a hook plate with distal‐only screws and minimal medial dissection to reduce mediastinal risk, achieving union and uneventful removal. Adjunctive teriparatide therapy may have contributed to union and therefore confounds interpretation of fracture healing; consequently, union cannot be attributed to the hook plate alone. Other salvage strategies reported in the literature include sternoclavicular spanning constructs, ligament reconstruction techniques, and custom or inverted plate configurations [7, 12, 13].

**Table 1:** Condensed clinical timeline (dates pre‐presentation; post‐op as POD). Index (POD #0) = 2024‐05‐08.

When	Key events and outcomes
2024‐03‐07	Local clinic first operation (unsuccessful).
2024‐04‐16	Local clinic second operation (unsuccessful).
2024‐04‐23	Presented to our hospital; SC joint intact; proximal comminution; distal fragment anterior–superior displacement.
POD # 0 (2024‐05‐08)	Our hospital surgery: removal of prior hardware; hook plate fixation; alignment restored.
POD # 3Mo	Follow‐up: progressing union; symptoms improved.
POD # 5Mo 2Wks (2024‐10‐28)	Implant removal; maintained union; return to daily activities without restriction.
POD # 11Mo 1Wk (2025‐04‐18)	Final outpatient follow‐up; union well maintained; VAS pain ≤ 1/10; full activity.

## 5. Conclusion

This case describes revision management of a complex medial‐end clavicle fracture after two failed prior fixation attempts. Hook plate fixation with distal‐only screw purchase restored alignment and radiographic union, with planned implant removal after healing. However, healing cannot be credited solely to the hook plate because adjunctive teriparatide may have influenced union. The absence of standardized functional outcome scores further limits interpretation, and conclusions drawn from a single case should not be generalized. Written informed consent was obtained from the patient for publication of this case report and the accompanying images.

A completed CARE checklist is provided as Supporting Information S1. The clinical course is summarized in Table 2 and depicted in a timeline figure (Figure 4).

**Table 2:** Postoperative rehabilitation protocol.

Phase/time	Immobilization	Range of motion	Activity restrictions/goals
0–2 weeks	Sling for comfort	Hand/wrist/elbow ROM; pendulum as tolerated	Wound care; avoid lifting/pushing/pulling
2–6 weeks	Wean sling as pain allows	Begin passive then active‐assisted shoulder ROM	No lifting > 1–2 kg; avoid cross‐body adduction
6–12 weeks	None routinely	Progress to active ROM; gentle stretching	Light ADLs; no heavy labor or contact activity
3–5 months	None	Full ROM; begin strengthening as tolerated	Gradual strengthening; avoid heavy lifting until radiographic healing
After confirmed union/after implant removal	None	Return‐to‐activity progression	Advance to unrestricted activity as symptoms allow

**Table 3:** Selected literature on medial‐end clavicle fractures (design, treatment, and outcomes).

Study (year)	Design/*N*	Treatment	Key outcomes	Complications
Asadollahi and Bucknill (2019) [11]	Systematic review, 220 adults	191 nonoperative; 29 operative	Nonunion ~5%; nonoperative generally favorable.	No intraoperative complications reported among operative cases.
Salipas et al. (2016) [2]	Retrospective, Level‐1 center, 68	Mostly initial non‐op; 2.9% delayed surgery for painful delayed union	Predominantly nonoperative; ASES ~80, SSV ~77.	In‐hospital mortality 4.4% (polytrauma cohort); one intra‐op vascular event without long‐term harm
Frima et al. (2020) [14]	Retrospective cohort, 14 (displaced)	Locking plates (e.g., VA‐LCP)	Locking‐plate; QuickDASH 0.81; SSV 96; NO nonunion.	One early revision with later infection; 8 implant removals
Kim et al. (2023) [10]	Retrospective, 18 (Edinburgh 1B)	Hook plate fixation	Hook‐plate; union 100% (~6.2 mo, CT); manubrial osteolysis ~33%; planned removal common.	Manubrial osteolysis 33.3%; recommend plate bending and routine removal

This is a narrative, nonsystematic summary intended to contextualize operative options; it is not a systematic review.

Note that studies were selected because they reported clinical outcomes for medial‐end clavicle fractures managed nonoperatively or with commonly used operative constructs (e.g., locking plates or hook plates). Outcome measures, follow‐up duration, and reported complications vary across reports, which limits synthesis. The table is provided to illustrate the range of approaches and outcomes; no direct comparisons are intended.

After confirming radiographic union, the implant was removed on 28 October 2024. At the last outpatient follow‐up on 18 April 2025, union was maintained and the patient had returned to full daily activity. Pain was minimal (VAS ≤ 1/10); standardized functional outcome scores (e.g., DASH/ASES) were not collected and are acknowledged as a limitation (Figures 5, 6, and 7).

Figure 5Follow‐up radiographs. (a) POD 3 months, AP. (b) POD 3 months, cephalad. (c) POD 5 months, AP. (d) POD 5 months, cephalad. (e) POD 5 months, axial CT.

**Figure:**
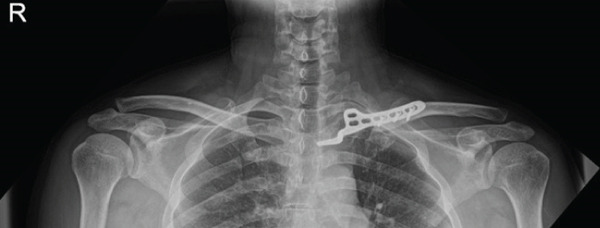
(a)

**Figure:**
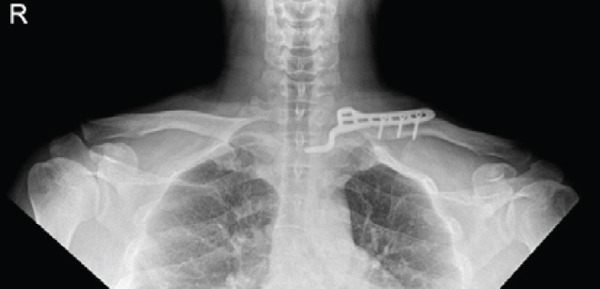
(b)

**Figure:**
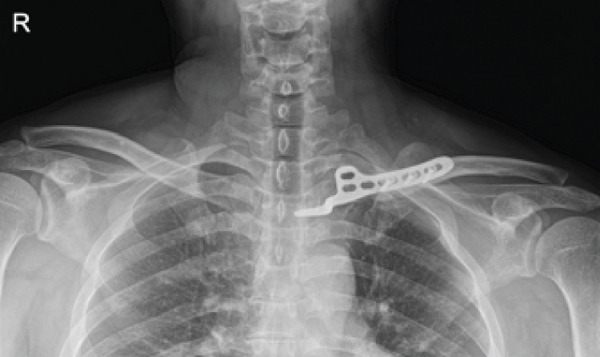
(c)

**Figure:**
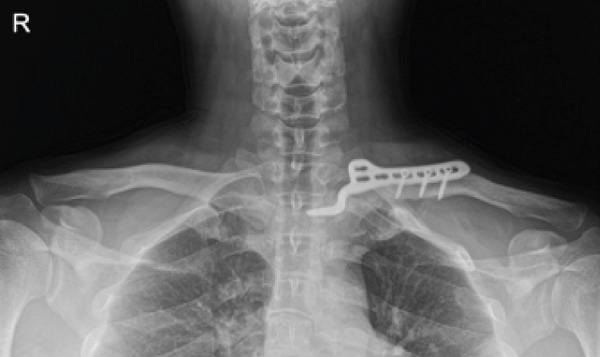
(d)

**Figure:**
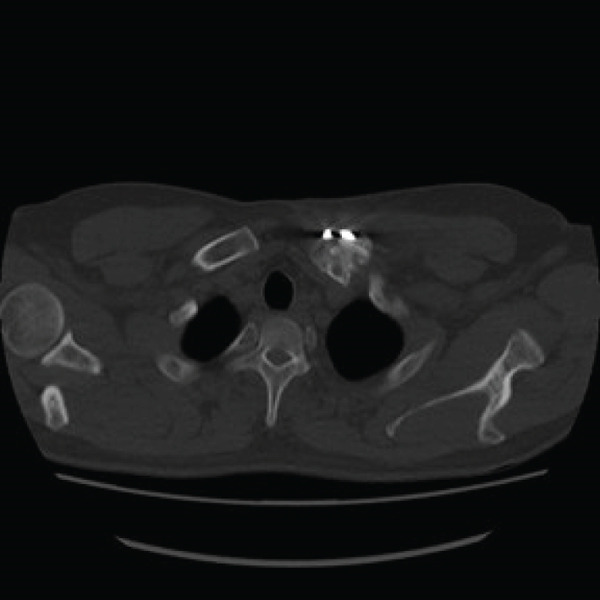
(e)

Figure 6Day of implant removal (2024‐10‐28). (a–b) AP/cephalad radiographs show maintained union.

**Figure:**
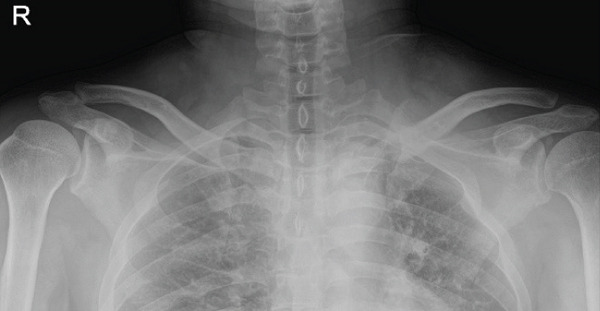
(a)

**Figure:**
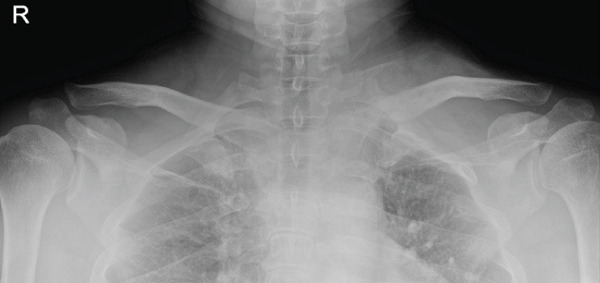
(b)

Figure 7Final outpatient follow‐up (2025‐04‐18). (a–b) AP/cephalad radiographs demonstrate maintained union.

**Figure:**
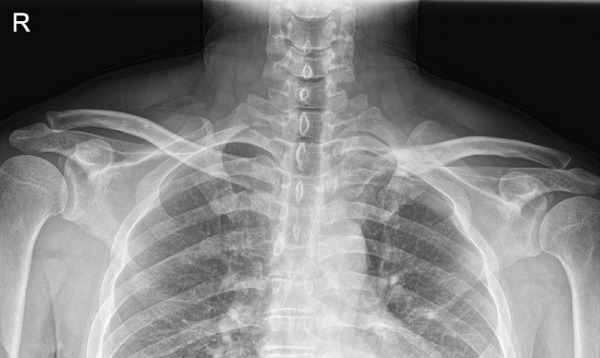
(a)

**Figure:**
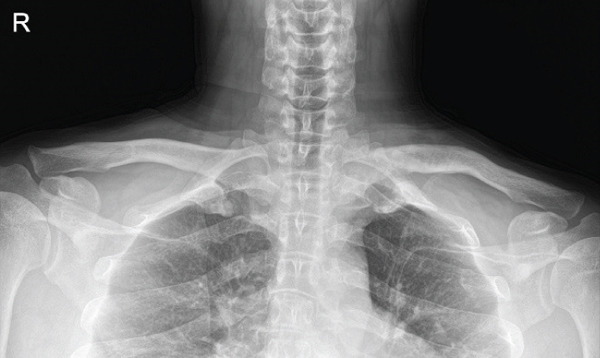
(b)

All radiographs and CT images were fully de‐identified prior to submission.

## Funding

No funding was received for this manuscript.

## Disclosure

This work was previously presented as a poster at the 67th Annual Congress of the Korean Orthopaedic Association, 2023

## Conflicts of Interest

The authors declare no conflicts of interest.

## Supporting information


**Supporting Information** Additional supporting information can be found online in the Supporting Information section. The following supplementary material is available online: Supporting Information S1: A completed CARE checklist.

## Data Availability

The data that support the findings of this study are available on request from the corresponding author. The data are not publicly available due to privacy or ethical restrictions.
