# Supplementary material for: Management of a Complex Medial‐End Clavicle Fracture With Hook Plate Fixation After Failed Prior Surgeries: A Case Report and Narrative Review of Surgical Options
Source: Case Rep Orthop. 2026 Apr 30;2026:5764986. doi: 10.1155/cro/5764986 (PMC13130850; doi:10.1155/cro/5764986)
Supplement: Supplementary file 1 — Supporting Information Additional supporting information can be found online in the Supporting Information section. The following supplementary material is available online: Supporting Information S1: A completed CARE checklist. [file CRO-2026-5764986-s001.docx]

**Supplementary Material S1. CARE Checklist (Completed)**

Manuscript: Management of a Complex Medial-End Clavicle Fracture with Hook Plate Fixation after Prior Fixation Failure.

The checklist below indicates where each CARE item is addressed in the manuscript.

| CARE item | What is reported | Where in manuscript |
| --- | --- | --- |
| Title | Key diagnosis/therapy and 'case report' in title | Title page |
| Keywords | Key terms related to condition and intervention | Title page / Keywords (if required by journal) |
| Abstract | Brief background, patient, intervention, outcomes, conclusion | Abstract |
| Introduction | Background and rationale | Introduction |
| Patient information | De-identified demographics, relevant history, comorbidities, risk factors | Case presentation |
| Clinical findings | Presenting symptoms, physical findings | Case presentation |
| Timeline | Chronological sequence of events from injury to follow-up | Table 2 and Figure 7 |
| Diagnostic assessment | Imaging and diagnostic reasoning; differential diagnoses and rationale | Case presentation (Diagnostic reasoning paragraph) |
| Therapeutic intervention | Surgical technique and adjunctive therapy; rationale | Operative details and postoperative course |
| Follow-up and outcomes | Clinical course, imaging follow-up, implant removal, final status | Operative details and postoperative course; Figures |
| Adverse events | Complications or absence thereof (as observed) | Operative details and postoperative course; Discussion |
| Discussion | Strengths/limitations, relevant literature context, rationale for conclusions | Discussion |
| Patient perspective | Patient-reported experience (if available) | Not reported (not available) |
| Informed consent | Consent for publication and images | Conclusion / Consent statement |
